# Conflict and competition between model-based and model-free control

**DOI:** 10.1371/journal.pcbi.1010047

**Published:** 2022-05-05

**Authors:** Yuqing Lei, Alec Solway

**Affiliations:** 1 Department of Psychology, University of Maryland-College Park, College Park, Maryland, United States of America; 2 Program in Neuroscience and Cognitive Science, University of Maryland-College Park, College Park, Maryland, United States of America; University College London, UNITED KINGDOM

## Abstract

A large literature has accumulated suggesting that human and animal decision making is driven by at least two systems, and that important functions of these systems can be captured by reinforcement learning algorithms. The “model-free” system caches and uses stimulus–value or stimulus–response associations, and the “model-based” system implements more flexible planning using a model of the world. However, it is not clear how the two systems interact during deliberation and how a single decision emerges from this process, especially when they disagree. Most previous work has assumed that while the systems operate in parallel, they do so independently, and they combine linearly to influence decisions. Using an integrated reinforcement learning/drift-diffusion model, we tested the hypothesis that the two systems interact in a non-linear fashion similar to other situations with cognitive conflict. We differentiated two forms of conflict: *action conflict*, a binary state representing whether the systems disagreed on the best action, and *value conflict*, a continuous measure of the extent to which the two systems disagreed on the difference in value between the available options. We found that decisions with greater value conflict were characterized by reduced model-based control and increased caution both with and without action conflict. Action conflict itself (the binary state) acted in the opposite direction, although its effects were less prominent. We also found that between-system conflict was highly correlated with within-system conflict, and although it is less clear a priori why the latter might influence the strength of each system above its standard linear contribution, we could not rule it out. Our work highlights the importance of non-linear conflict effects, and provides new constraints for more detailed process models of decision making. It also presents new avenues to explore with relation to disorders of compulsivity, where an imbalance between systems has been implicated.

## Author summary

A number of studies have framed goal-directed and habitual decision making from the perspective of different reinforcement learning algorithms (“model-based” and “model-free”), and further suggested that they are supported by separate though potentially overlapping systems. However, there has been little work to understand how the different systems work together. By design, they will sometimes disagree on the identity of the best action, and even when they agree, they will assign different values to the actions. Despite this, the end result is a single behavioral output. The issue of how the two systems interact and compete draws parallels to the existing literature on cognitive control, where a central question has been how more ‘costly’ cognitive resources should be deployed in the presence of decision conflict (here, the goal-directed system is more computationally ‘expensive’). Across four datasets, we found that the influence of the goal-directed system was reduced as a function of conflict between systems, and in addition, responses overall were more cautious. Our results provide new constraints for process models of decision making, and suggest new research directions for questions related to psychopathology and disorders of compulsivity in particular, where an imbalance between the two systems has previously been implicated.

## Introduction

The last decade and a half has seen an explosion of work formalizing goal-directed decision making using the language of reinforcement learning [1–9] (RL), adding to a previous literature describing habitual decisions using this same framework [10, 11]. Habitual control has been aligned with ‘model-free’ RL, which consists of algorithms for constructing stimulus–value or stimulus–response associations based on experience. Goal-directed control has been aligned with ‘model-based’ RL, which includes quite similar algorithms that learn a model of the world in the form of a state transition and reward function, and use this model to make decisions. This work has largely assumed that the two decision systems described by these algorithms operate in parallel, and critically, with the exception of a few individual studies that have posited specific more complicated relationships [2, 5, 12], that the outputs of these two systems are computed independently and then combined linearly to contribute to choice [4, 6, 7, 13, 14]. From a computational perspective, each system assigns a weight to each option under consideration—weights which may differ due to the different assumptions made by the two classes of algorithms—and these weights are then simply added together before being transformed by a link function that maps weighted value to choice. (The link function itself may be nonlinear and is usually the softmax function, however, it is applied at a later step after combining the output of the two systems.) This same reinforcement learning framework has also been used to investigate impairments in decision making associated with psychopathology, including binge eating, substance use, and obsessive-compulsive disorder [15–17], and to test for differences under stress and cognitive load [18, 19]. Here too, the assumption has been that the contributions of the two systems sum linearly, and that potential impairments influence each system separately. In particular, these traits and situations have been linked to reduced model-based control.

By design, the values assigned to options by the two systems usually diverge. This can happen in two ways: they can disagree about value (we will refer to this as *value conflict* throughout), and they can disagree about which option is better (we will refer to this as *action conflict* because the options are usually tied to specific responses). These possibilities are of course not mutually exclusive. The two systems may agree on which option is more valuable, but disagree about the extent, and likewise, when they disagree about which option is more valuable, the degree of disagreement can be measured on a continuum in terms of differences in predicted values.

Disagreement, or conflict, between model-based and model-free decision systems draws parallels to the wider literature on conflict monitoring and control [20, 21]. The Stroop task is a canonical exemplar task from this line of work [22, 23]. In the (standard) Stroop, participants have to name the font color of a word while ignoring the word itself which may represent a different color, or vice versa. Incongruence in the stimulus results in conflict, which behaviorally manifests as increased reaction time, though these effects are significantly larger when having to name the font color while ignoring the meaning of the word. In contrast, font color does not significantly interfere with word reading [23]. In order to explain these effects, models have suggested that some responses are more prepotent and automatic, but that they can be overridden by top-down control employed as a result of conflict monitoring and task demands [20, 22]. All together, this results in an interactive non-linear effect: without conflict, the prepotent response dominates based on the degree of its default intensity, while this intensity is subdued in the presence of conflicting demands.

In the current work, we used this basic prediction as a starting point for investigating the effects of value and action conflict between model-based and model-free control. In this context, the model-free system, by relying on cached values rather than more costly rollouts of potential outcomes, may represent a generically more automatic decision system. Of course it should be noted that it is not clear that models which can successfully explain conflict between individual responses will necessarily translate directly to explain conflict between entire decision systems. In addition, which system represents a more prepotent mode of operation is also not without debate [5]. Nevertheless, with this background and potential complexity of the answer in mind, we asked about the intensity of model-based and model-free control under different degrees of value conflict, both with and without action conflict.

To ask these questions, we used the popular ‘two-step’ task designed to distinguish the control employed by the two systems [4, 7]. Given the potentially loose a priori constraints on predictions described above, we tested the replicability of the results across four datasets, using data previously published by us and previously collected but unpublished, as well as publicly available data collected by others [7, 14]. The latter also allowed us to ask these questions in a different version of the task, which is in many ways similar but was designed such that employing more model-based control results in a larger payoff compared to the original version [7]. We expected the strength of model-based control to be larger, or the strength of model-free control to be lower, as a function of the disagreement between the two systems (value conflict) when they favored different actions (i.e. in the presence of action conflict; when they agree on the best course of action, there appears little reason to modulate control). To briefly preview our results because they go against this prediction, we found consistent evidence that the strength of model-based control was reduced as a function of value conflict, both on trials with and without action conflict. Model-free control was simultaneously reduced on trials with action conflict independent of the degree of disagreement, but this reduction was not significantly different from the corresponding change in model-based control.

In addition to asking about how the degree of influence of each system changed as a function of conflict, we also asked how inter-system conflict related to overall decision caution. We did this by analyzing the data using an integrated reinforcement learning/drift-diffusion model that accounts for both learning and decision making, and that separates different components which make up the decision process. The drift-diffusion model is part of a broader class of evidence integration models which propose that a decision is made by incrementally collecting information over time until reaching a threshold [24, 25]. Such models are able to account not only for choice data, but also reaction time and confidence, and can help link behavioral findings to their neural implementation [26, 27]. They have been applied across a range of cognitive domains, including to reward-based decision making [28–31], where it is assumed that values are not directly accessible, but are instead sampled in a noisy fashion [32]. With some exception [33, 34], these models have not yet been widely applied to model-based reinforcement learning and the two system RL-based account of decision making. In addition to providing a more complete picture of the process of deliberation, evidence integration models parcellate it into distinct components, and include separate parameters that represent the average preference for one option over another (here, for each decision system) and decision impulsivity/caution. We took advantage of this ability and asked about the relationship between inter-system conflict and each of these quantities.

A priori we might expect increased caution specifically during trials with action conflict: assuming model-based control would then be dominant but the overall decision preference signal weak because the systems favor different actions, increased caution would allow additional evidence to be accumulated according to the preferences of the model-based system. Some support for the idea that action conflict is associated with increased caution comes from studies in both the perceptual and reward-based domains, although it should be noted that in the case of reward-based decisions, a more consistent effect has been increased caution moderated by specific neural signatures—including theta-band medial prefrontal cortex activity and activity in the subthalamic nucleus—rather than a main effect [28, 29, 35]. To briefly preview these results, we found that value conflict was related to more caution both in the presence and absence of action conflict. There was some evidence that the binary state of action conflict was associated with a change in the opposite direction—decreased caution.

Finally, value conflict between systems is likely correlated with value conflict within systems (i.e. the differences in value between the two choices). We assessed the extent to which within-system conflict could be disambiguated from between-system conflict, and whether it influenced decision making above and beyond its standard linear contribution.

## Methods

### Datasets

We included four datasets (Table 1) in this study, three from previously published work [7, 14], and one previously collected but unpublished. The data in [14] and the unpublished dataset were collected in-person in the lab, while the other two were collected on Amazon Mechanical Turk [7]. In all datasets, participants who had more than 20% trials with reaction time < 0.2*sec* or a missing choice in either stage were excluded from the analysis. Three of the datasets were based on the original version of the two-step task, and one based on a newer version in which model-based control has been shown to pay off significantly more [7]. Both task variants are described at a high level in the main text, and we refer readers to the previously published work on these datasets for additional details [7, 14]. The one unpublished dataset was based on the original task variant. Although similar experimental procedures were followed in performing this experiment, we describe them next for completeness.

**Table 1:** Sources and sample sizes of the four datasets.

**Original task**	Solway et al. [14]	Kool et al. [7]	Unpublished data
Collected sample size	n = 119	n = 206	n = 92
After exclusion	n = 115	n = 175	n = 84
**Newer version**	Kool et al. [7]		
Collected sample size	n = 199		
After exclusion	n = 179		

### Ethics statement

For the previously unpublished dataset, experimental procedures were approved by the Princeton University Institutional Review Board. Written informed consent was obtained in accordance with the approved procedures.

### Previously unpublished dataset details

Ninety-two participants from the Princeton University campus completed the experiment. Each trial had two stages. The first stage involved a binary decision between the same two stimuli (randomly positioned on the left/right side of the screen), and the selected choice probabilistically led to one of two other states. Transition probabilities were 0.7/0.3 and 0.3/0.7 as in previous work. When participants selected a choice from the first-stage choice pair, the chosen stimulus was highlighted. Then, the selected stimulus moved to the top, the non-chosen stimulus disappeared, and the resulting second-stage choice pair appeared below. Each second-stage state also featured an independent binary decision that led to a probabilistic 0/1 outcome. The reward rate of each second-stage choice changed independently according to a Gaussian random walk with reflecting boundaries at 0.25 and 0.75 and a standard deviation of 0.025. There was a two second deadline for both decisions. Participants completed a tutorial session with practice trials, followed by 201 trials of the experiment. They also completed several questionnaires, which were not analyzed here.

### Reinforcement learning

The first part of our model was based on previous work with this task [4, 7, 13, 14]. Action values for each state and action (stimulus), *Q*(*s*, *a*), were computed using separate model-based and model-free learning algorithms. The model-free system updated values from experience using SARSA(λ) [36]. The update for the value of the action chosen at the first stage on trial *t* was based on both the value of the second-stage action and the eventual outcome (*rew*) at the end of the trial:

$$\begin{array}{cc} Q_{mf}{\left(state_{stage1},choice_{stage1}\right)}_{t}^{\prime }= & Q_{mf}{\left(state_{stage1},choice_{stage1}\right)}_{t-1}+\\ & \alpha \cdot [Q_{mf}{\left(state_{stage2},choice_{stage2}\right)}_{t-1}-\\ & \;Q_{mf}{\left(state_{stage1},choice_{stage1}\right)}_{t-1}]. \end{array}$$ (1)

$$\begin{array}{cc} Q_{mf}{\left(state_{stage1},choice_{stage1}\right)}_{t}= & Q_{mf}{\left(state_{stage1},choice_{stage1}\right)}_{t}^{\prime }+\\ & \lambda \cdot \alpha \cdot [rew-Q_{mf}{\left(state_{stage2},choice_{stage2}\right)}_{t-1}]. \end{array}$$ (2)

The value of the action chosen at the second stage was similarly updated according to:

$$\begin{array}{cc} Q_{mf}{\left(state_{stage2},choice_{stage2}\right)}_{t}= & Q_{mf}{\left(state_{stage2},choice_{stage2}\right)}_{t-1}+\\ & \alpha \cdot [rew-Q_{mf}{\left(state_{stage2},choice_{stage2}\right)}_{t-1}]. \end{array}$$ (3)

Values for non-selected choices decayed towards the initial *Q* value:

$$\begin{array}{c} Q{\left(s,a\right)}_{t}=Q{\left(s,a\right)}_{t-1}+\alpha \cdot \left[Q_{initial}-Q\left(s,a\right)\right]. \end{array}$$ (4)

*Q*_*initial*_ was set to the midpoint reward in each version of the task: (1 + 0)/2 = 0.5 in the original version and (−4 + 5)/2 in the newer version. Further, rewards were re-scaled in the second task version by dividing them by 9 in order to put the differences between the Q-values on a similar scale as in the first task version. (The largest difference in Q-values in the second version was 5 − −4, while in the first version it was 1 − 0.)

The model-based system computed separate first-stage action values according to:

$$\begin{array}{c} Q_{mb}\left(state_{stage1},choice_{stage1}\right)=\sum_{s^{\prime }}\;p\left(s^{\prime }|state_{stage1},choice_{stage1}\right)\cdot \underset{a^{\prime }}{\text{max}}\;Q_{mb}\left(s^{\prime },a^{\prime }\right). \end{array}$$ (5)

It shared second-stage action values with the model-free system. In the original task version, *p*(*s*′|*state*_*stage*1_, *choice*_*stage*1_) was set to one of the true underlying probabilities (0.7 or 0.3) depending on the transitions experienced by the participant up until that trial. If the number of times selecting action 1 and ending up in state 2 plus the number of times selecting action 2 and ending up in state 3 was more than than the sum of the opposite transitions, *p*(*s* = 2|*s* = 1, *a* = 1) and *p*(*s* = 3|*s* = 1, *a* = 2) were set to 0.7, and *p*(*s* = 2|*s* = 1, *a* = 2) and *p*(*s* = 3|*s* = 1, *a* = 1) were set to 0.3. Otherwise, the opposite assignments were made. In the second task version, transitions were deterministic and transition probabilities for a pair of first-stage stimuli were set to their veridical values as soon as a transition for one of them was observed. On the very first trial of each pair, they were set to 0.5. Note that in the original task version the first-stage state was always the same, while in the second version there were two possible first-stage states (the two rockets pairs). In the second stage of the task, both versions had two second-stage states, but while in the original each state had two choices, in the second version each state had only one “choice”.

### Action selection and model fitting

Model fitting was performed using a two step procedure. During the first step, action values were mapped to choice probabilities using the logistic (softmax) function:

$$\begin{array}{l} P\left(state_{stage1},choice_{1}\right)=\\ \;\;\;\;logistic(\beta_{mb}\cdot \left[Q_{mb}\left(state_{stage1},choice_{1}\right)-Q_{mb}\left(state_{stage1},choice_{2}\right)\right]+\\ \;\;\;\;\;\;\;\beta_{mf}\cdot \left[Q_{mf}\left(state_{stage1},choice_{1}\right)-Q_{mf}\left(state_{stage1},choice_{2}\right)\right]+\\ \;\;\;\;\;\;\;\beta_{rep}\cdot rep) \end{array}$$ (6)

where *rep* was 1 if neither deadline was missed on the previous trial and *choice*_1_ was selected at the first-stage, and −1 if *choice*_2_ was selected. Similarly, for the original version of the task which had a second-stage decision,

$$\begin{array}{cc} P(state_{stage2},choice_{1})= & \\ \;\;\;\;\;\;logistic(\beta_{2}\left[Q_{mf}\left(state_{stage2},choice_{1}\right)-Q_{mf}\left(state_{stage2},choice_{2}\right)\right]). \end{array}$$ (7)

Recall that *Q*_*mf*_ = *Q*_*mb*_ at the second stage. The following were free parameters: *α*, λ, *β*_*mb*_, *β*_*mf*_, *β*_*rep*_, and *β*_2_. Models were fit within a multi-level Bayesian framework implemented in Stan [37]. Multi-level models allow for more accurate parameter estimates by taking the entirety of the data into account together [38]. Wide unbiased priors were used for all group-level parameters: *α*^*μ*^, *α*^*σ*^, λ^*μ*^, λ^*σ*^, $\beta_{mb}^{\mu }$, $\beta_{mb}^{\sigma }$, $\beta_{mf}^{\mu }$, $\beta_{mf}^{\sigma }$, $\beta_{2}^{\mu }$, $\beta_{2}^{\sigma }$, $\beta_{rep}^{\mu }$, $\beta_{rep}^{\sigma }$ each had a prior of *N*(0, 20). Subject-level parameters had Gaussian priors constrained by the group-level parameters: *α* ∼ *N*(*α*^*μ*^, *α*^*σ*^), λ ∼ *N*(λ^*μ*^, λ^*σ*^), and so on for the remaining parameters. Subject-level *α* and λ parameters were transformed to the 0–1 range using a logistic function. Standard deviation parameters were constrained to be greater than or equal to 0.

After fitting this version of the model, we extracted the median *α* and λ estimates for each participant, and used them to compute Q-values for each participant/trial. We then performed a second round of inference to fit the drift-diffusion model, including conflict effects, based on these Q-values. The drift-diffusion model was fit to both choices and reaction times. Our overall analysis was thus quasi-Bayesian in that we used a two step model fitting procedure and point estimates for *α* and λ rather than their full posterior distribution. This approximative scheme was necessary for a technical reason. Stan and the No U-Turn Sampler provide an efficient method for obtaining posterior samples from complex multi-level models. However, efficient sampling requires that the (unnormalized) posterior is differentiable. Both the sudden change at zero in the definition of action conflict (see Eq 11) and the absolute value function in the definition of value conflict (Eq 10) violated this requirement, requiring us to adopt the two step process. We tested the validity of this approach in two ways, as reported further below and in the *Results*. First, we simulated synthetic data and tested the procedure’s ability to recover known parameter values. Second, we re-ran all of the analyses using a simplified model that could be fit in a single step, and found similar results.

During the second step of the model fitting procedure, we fit a simple drift-diffusion decision process (without across-trial variance) to each of the first and second stage decisions. Drift rate (*v*_1_) and boundary separation (*a*_1_) for the first stage were defined according to Eqs 12–14 in the main text below, which include conflict terms as regressors. The starting preference was defined as:

$$\begin{array}{c} s_{1}=logistic\left(s_{rep}\cdot rep\right), \end{array}$$ (8)

ranging from the lower to the upper boundary. In the drift-diffusion model, bias may be implemented in two ways: in terms of the drift rate and in terms of the starting preference. Eq 13 specifies how choice perseveration is implemented in terms of drift rate, and Eq 8 specifies how it is implemented in terms of the starting preference. To our knowledge, previous work has not made this distinction. Drift rate for the second stage was defined according according to:

$$\begin{array}{c} v_{2}=v_{2,base}\cdot \left[Q_{mf}\left(state_{stage2},choice_{1}\right)-Q_{mf}\left(state_{stage2},choice_{2}\right)\right], \end{array}$$ (9)

and the boundary was a free parameter that lacked regressors. The starting preference at the second stage was fixed to 0.5. The drift-diffusion model also has a fourth free parameter, *τ*, representing non-decision time, and this was shared between the two decision stages. Note that although including across-trial variance in parameters allows the model to explain a larger range of main effects regarding accuracy and reaction time [25], other work has shown that excluding them can result in improved model fit if these effects and parameters are not of interest [39]. We excluded these parameters for this reason. In all, the following were free parameters: *v*_*mb*,*base*_, *v*_*mb*,*conflict*,*value*_, *v*_*mb*,*conflict*,*action*_, *v*_*mb*,*conflict*,*interaction*_, *v*_*mf*,*base*_, *v*_*mf*,*conflict*,*value*_, *v*_*mf*,*conflict*,*action*_, *v*_*mf*,*conflict*,*interaction*_, *v*_*rep*_, *a*_1,*base*_, *a*_*conflict*,*value*_, *a*_*conflict*,*action*_, *a*_*conflict*,*interaction*_, *s*_*rep*_, *v*_2,*base*_, *a*_2_, and *τ*. Parameters were hierarchically defined similar to the first step of the model fitting procedure described above, with *N*(0, 20) priors on the group-level mean and standard deviation for most parameters (see below), and Gaussian priors with the corresponding group level mean and standard deviation for the subject-level parameters. There were three exceptions. First, the mean and standard deviation for conflict-related drift rate effects had *N*(0, 200) priors. These parameters are on a larger scale because they multiply an effect that is itself a result of multiplying two small numbers: the difference in Q-values between actions and inter-system value conflict. Second, the mean non-decision time had a *N*(0.5, 1) prior and the standard deviation across subjects had a *N*(0, 1) prior. Third, $a_{1,base}^{\mu }$ and $a_{2}^{\mu }$ had *N*(1, 20) priors, biased positive because boundary separation must be positive. The group level parameters for base boundary separation along with their subject-level counterparts, the group and subject-level parameters for non-decision time, and all standard deviation parameters were constrained to be greater than or equal to 0.

As described in the *Results* below, we also fit a second similar model in which non-linear effects were due to conflict between action values within each decision system rather than between systems. The same priors were used for the corresponding parameters in this model.

We tested the overall model fitting procedure in two ways. First, we simulated synthetic data and used the same process to recover parameters from the synthetic datasets. This was done for each experiment and model we analyzed, each time generating many datasets based on the participant and trial numbers in the empirical data (see Results and Figs A-D in S1 Text). Second, to obtain corroborating evidence, we also analyzed reduced versions of both models which could be fit in a single pass, and which made use of the full posterior for *α* and λ. The reduced model for between-system conflict did not distinguish action conflict, and value conflict for both models was defined based on the squared differences of their conflict terms instead of the absolute values of these differences (see Eqs 10, 15 and 16).

For all models, Markov Chain Monte Carlo was performed for each step of the model fitting procedure with three chains. Each chain was run for 10,000 (4,000 for the reduced models) total iterations, using the first 1,000 for warmup. The Gelman-Rubin $\hat{R}$ statistic was computed and ensured to be less than 1.1 for all variables. We treated an effect as significant if the parameter’s central 95% credible interval excluded 0.

## Results

### Experimental tasks

We asked these questions in the context of the widely used ‘two-step task’ [4]. We used data from two of our previous studies, one published [14] and another previously unpublished, and two publicly available datasets collected by another group [7]. The latter study included both a run of the original version of the task and a newer version in which the payoff for increased model-based control was more substantial (see also [40], who first documented the lack of incentive for being model-based in the original task). The inclusion of both task versions allowed us to further test the replicability of the results as well as their generalizability.

**Fig 1:**
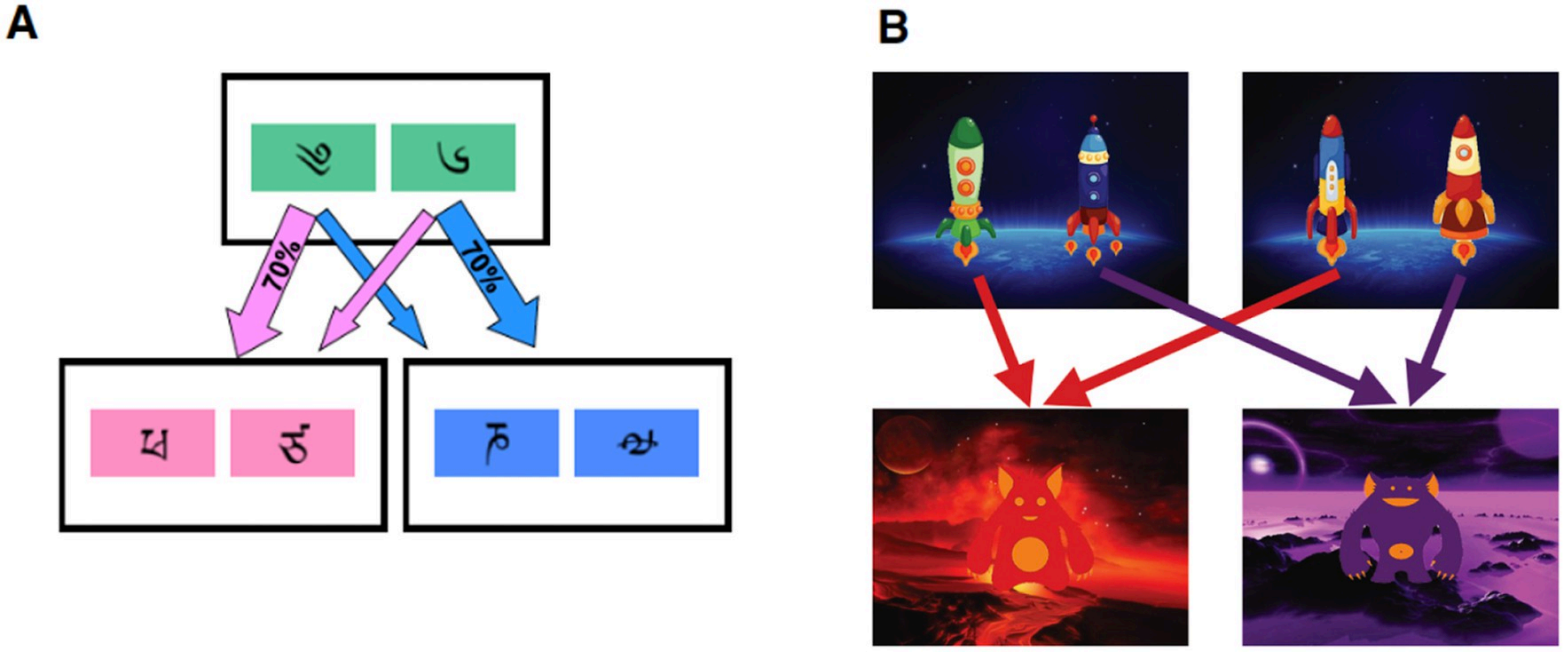
A. Trial structure in the original version of the two-step task. A first-stage choice between two options probabilistically led to a second-stage choice. Each second-stage choice had a randomly varying probability of paying a point or paying nothing. Figure from “Model-based influences on humans’ choices and striatal prediction errors” [4]. https://doi.org/10.1016/j.neuron.2011.02.027. Used under CC BY 3.0 (https://creativecommons.org/licenses/by/3.0/). B. Trial structure in a newer version of the two-step task where model-based control pays off to a greater degree. Each trial started with one of two pairs of stimuli which participants selected between. The choice led deterministically to one of two second-stage states, with payoffs that varied over the course of the experiment. Figure from “When does model-based control pay off?” [7]. https://journals.plos.org/ploscompbiol/article/figure?id=10.1371/journal.pcbi.1005090.g015. Used under CC BY 4.0 (https://creativecommons.org/licenses/by/4.0/).

Both versions are well-known, but we briefly describe them for ease of reference. In the standard version [4] (Fig 1A), participants make two choices on each trial. The first stage includes two options, each probabilistically leading to one of two second-stage states. Each second stage state also includes two options, leading to a probabilistic reward (0 or 1 point). The probability of reward for each second-stage option follows an independent Gaussian random walk with reflecting boundaries. When computing the values of the first-stage options, the model-free system is sensitive only to the reward or lack thereof experienced on each trial, ignoring the transition structure from the first to the second stage (cf. Fig 1A). In contrast, the model-based system takes the transition structure into account.

Although numerous studies have shown that people employ model-based control in this version of the task [4, 7, 14, 18, 19], increasing such control does not yield significantly more reward, and this motivated the development of an alternative version where increased model-based control does result in an additional payoff [7]. In this version (Fig 1B), the first stage starts with one of two pairs of options. Each option deterministically leads to one of two second-stage states which deliver reward in the range -4 to 5, with each amount evolving according to a Gaussian random walk. Here too, taking the transition structure between the first and second stage into account, versus ignoring this information, leads to different predictions about action values at the first stage over the course of the experiment.

### Definition of conflict and modeling of deliberation

Our analysis began by computing the value of each state and action, *Q*(*s*, *a*), separately according to the model-based and model-free systems, using standard methods employed in previous work [4, 7, 13, 14]. Details are provided in the *Methods*. Based on these values, we defined value conflict at the first stage (the two systems do not differ at the second stage) as:

$$\begin{array}{cc} conflict_{value}=| & (Q_{mb}\left(s,choice_{1}\right)-Q_{mb}\left(s,choice_{2}\right))-\\ & (Q_{mf}\left(s,choice_{1}\right)-Q_{mf}\left(s,choice_{2}\right))|. \end{array}$$ (10)

We defined action conflict as:

$$\begin{array}{cc} m= & (Q_{mb}\left(s,choice_{1}\right)-Q_{mb}\left(s,choice_{2}\right))*\\ & (Q_{mf}\left(s,choice_{1}\right)-Q_{mf}\left(s,choice_{2}\right)),\\ conflict_{action}= & \left\{ \begin{array}{cc} 0 & m\geq 0\\ 1 & m<0. \end{array}\right. \end{array}$$ (11)

Eq 10 defines a continuous measure of inter-system conflict that captures how much the systems (dis)agree about the difference in value between the two actions at the first stage. We call this *value conflict*. Eq 11 defines a binary measure, which we call *action conflict*, that captures whether or not the two systems agree on the identity of the best action. Our interest was primarily in tracking conflict in a continuous fashion, and as described below, we asked questions about value conflict and its interaction with action conflict.

We used the drift-diffusion model [24, 25] to map action values, including conflict effects, to choice. The drift-diffusion model describes decisions between two options. The relative amount of evidence between the options changes on average at a set rate (drift rate, a free parameter), but the exact trajectory is noisy. A decision is made when the relative evidence reaches one of two decision boundaries, whose distance translates into the level of impulsivity of the decision (and is also a free parameter). Other free parameters include the initial evidence before the decision begins, and non-decision time. The use of the drift-diffusion model allowed model fitting to be constrained by reaction time in addition to choice, and for us to ask about the effects of conflict on decision caution/impulsivity separately from the effects on the strength of each decision system. We set the upper boundary to be “choice 1” and the lower boundary to be “choice 2”, arbitrarily defined (all equations can be changed to swap these associations without affecting the results). The model-based and model-free decision systems influenced decision making through changes in drift rate. We defined the strength of the model-based system as:

$$\begin{array}{cc} v_{mb}=v_{mb,base}+ & v_{mb,conflict,value}\cdot conflict_{value}+\\ & v_{mb,conflict,action}\cdot conflict_{action}+\\ & v_{mb,conflict,interaction}\cdot conflict_{value}\cdot conflict_{action}, \end{array}$$ (12)

with a similar definition for the strength of the model-free system. Because *conflict*_*action*_ is dummy coded, this definition divides the continuous effects of value conflict into two groups: trials with and without action conflict. The full drift rate for the first-stage decision was then defined to be:

$$\begin{array}{cc} v_{1}= & v_{mf}\cdot \left[Q_{mf}\left(s,choice_{1}\right)-Q_{mf}\left(s,choice_{2}\right)\right]+\\ & v_{mb}\cdot \left[Q_{mb}\left(s,choice_{1}\right)-Q_{mb}\left(s,choice_{2}\right)\right]+\\ & v_{rep}\cdot rep, \end{array}$$ (13)

where *rep* was set to 1 when action 1 was selected on the previous trial and −1 when action 2 was selected. *v*_*rep*_ represents a continuous bias in drift rate in the same (or opposite if negative) direction as the previous choice, regardless of the reward received. In addition, as noted we also allowed conflict to affect the separation between the decision boundaries representing the two options:

$$\begin{array}{cc} a_{1}=a_{1,base}+ & a_{conflict,value}\cdot conflict_{value}+\\ & a_{conflict,action}\cdot conflict_{action}+\\ & a_{conflict,interaction}\cdot conflict_{value}\cdot conflict_{action}. \end{array}$$ (14)

It is important to note that although the drift-diffusion model is sometimes taken to describe the actual algorithm the brain uses during deliberation (e.g. [26]), here we are using it as a descriptive statistical model. Eq 12 says that the strength of the model-based controller can change as a function of conflict (and similarly for the model-free controller), but we do not make strong claims about the specific algorithm or implementation by which this might happen. As we note in the *Discussion*, answering this question requires a better understanding of the specific way in which the brain performs model-based rollouts, which is an open area of research.

### Model fitting

We fit the above model using an approximate hierarchical Bayesian framework, described in *Methods*. In order to both test the model fitting procedure and ensure that we were able to recover the parameters of interest given the number of parameters in the overall model, we simulated and fit synthetic data with the same number of participants and trials present in each experimental dataset. These results are shown in Figs A-D in S1 Text. Parameter recovery was overall reasonable. Estimates of the effects of conflict on drift rate appeared unbiased (to be either more positive or more negative) across simulations. Despite the fact that priors at the group level were very broad, there was evidence that estimates were symmetrically pushed towards the prior mean of zero, suggesting that these effects were weakly constrained. Priors can help guard against spurious results under such circumstances. In contrast, some bias was present in the boundary separation parameters, with a potential tradeoff between the baseline and the effect of value conflict without action conflict (in the original task version), as well as the baseline model-free drift rate (also in the original task version) and the baseline model-based drift rate (in the newer task version). Biases for value conflict effects in the newer task version went in the opposite direction. These results could not explain the full pattern of boundary effects reported below, which were consistent across task versions and with and without the presence of action conflict.

### The strength of model-based control was negatively correlated with value conflict

**Fig 2:**
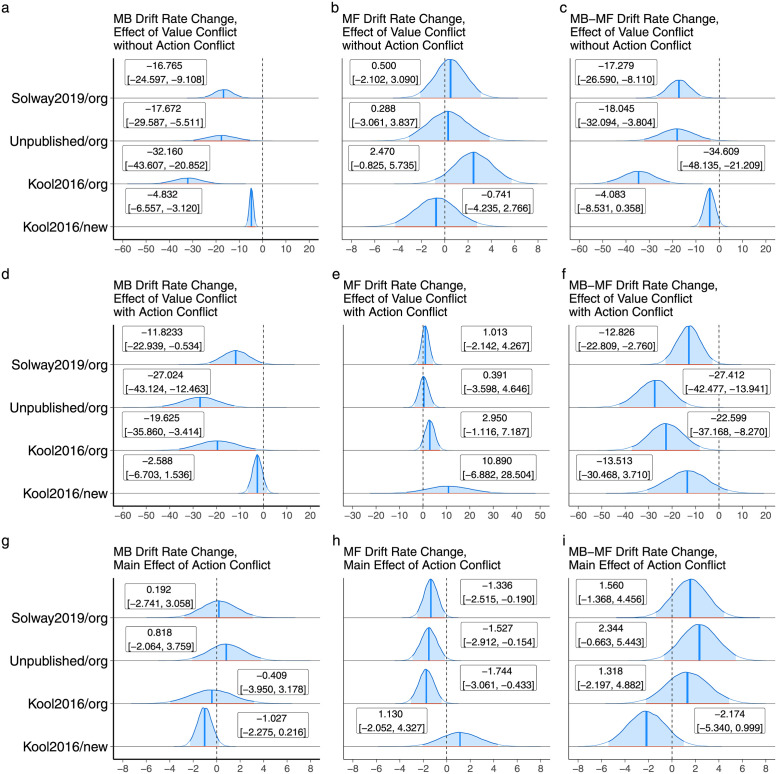
First row: The effect of value conflict in the absence of action conflict on the strength of model-based and model-free control in all four datasets. Second row: The same as the first row, but for trials with action conflict. Third row: Main effect of action conflict on model-based and model-free control.

Empirical results are reported in terms of the marginal posterior distributions of the parameters of interest, as well as their medians and central 95% credible intervals. The latter quantitative information is shown in the figures rather than embedded in the text for ease of reference. Our primary questions about decision system control regarded the parameter *v*_*mb*,*conflict*,*value*_, the sum *v*_*mb*,*conflict*,*value*_ + *v*_*mb*,*conflict*,*interaction*_, and their model-free equivalents. Respectively, these represent the effects of the continuous measure of conflict (value conflict) without and with action conflict (i.e. separately for trials where the two systems agree/disagree on the identity of the best action). Fig 2 displays these results. Without action conflict, the strength of the model-based controller was significantly reduced as a function of value conflict across all four datasets, including the three datasets based on the original version of the task, and the dataset based on the newer version (Fig 2A). No significant changes were seen in the strength of the model-free controller in any dataset (Fig 2B), and the differences between the effects on the two systems were significant in three of four datasets, with a marginally significant difference in the fourth (Fig 2C). With action conflict, the strength of model-based control was significantly reduced as a function of value conflict in the three datasets based on the original task version, but not the newer version where increased model-based control pays off (Fig 2D). No significant differences were seen in the strength of model-free control (Fig 2E), and the differences between the changes for the two systems were significant for the original task variant where we saw an effect on model-based control (Fig 2F).

Fig 2G–2I shows the main effects of action conflict (the dummy coded variable in Eq 11). We had no particular hypothesis about this term as it does not take the *degree* of conflict into account, but we present these results for completeness. There was evidence of a reduction in model-free strength (Fig 2H), but these effects were not significantly different from (themselves non-significant) changes in model-based strength (Fig 2I).

**Fig 3:**
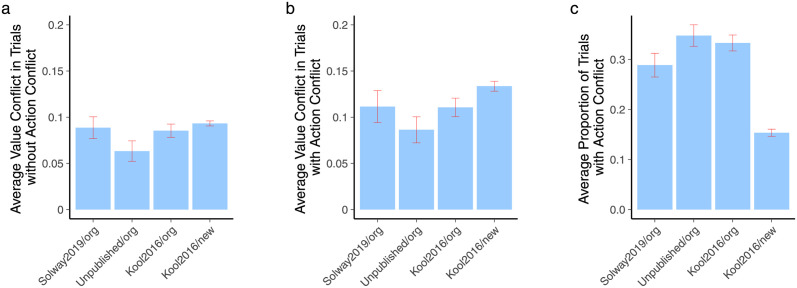
A. Average value conflict for trials without action conflict. Note that the values for the newer task version (Kool2016/new task) were normalized to put them on the same scale as the original task version. See Methods. B. Average value conflict for trials with action conflict. C. Average proportion of trials with action conflict.

We also compared average value conflict (without and with action conflict) and the average percentage of trials with action conflict *across datasets and tasks*. These are plotted in Fig 3. Note that the raw scale of values in the newer task variant is different because rewards range from -4 to 5 instead of being 0/1 as in the original. Here and in all analyses (see Methods) we normalized reward in this task by dividing it by 9. Value conflict was similar across the four datasets. However, the proportion of trials with action conflict was lower in the newer task (Fig 3C).

Finally, because our model fitting procedure required two separate steps (see Methods), in addition to testing parameter recovery from simulated data, we also stress-tested the results using a simplified model that allowed for full Bayesian inference in a single model-fitting step. In the reduced model, value conflict was defined based on the squared difference between the systems’ predictions instead of the absolute value (see Eq 10), and action conflict was not distinguished. The results of this model were similar to the main results (Fig E in S1 Text).

### Decision boundary separation was positively correlated with value conflict

**Fig 4:**
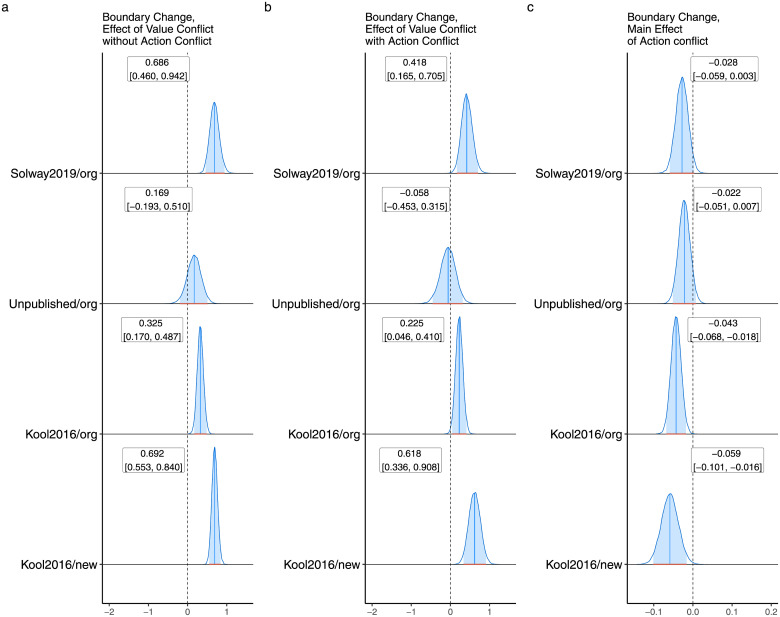
A. The effect of value conflict in the absence of action conflict on decision boundary separation. B. The same as (A), but for trials with action conflict. C. Main effect of action conflict on decision boundary separation.

Fig 4 displays the effects of inter-system conflict on the separation between decision boundaries. Boundary distance increased as a function of value conflict on trials both with and without action conflict in three out of four datasets. The presence of action conflict (the dummy coded variable) was associated with a change in the opposite direction, but the latter effect was less prominent overall and “significant” in only two datasets. Fig F in S1 Text shows the corresponding results for the reduced model that could be fit in a single step, also demonstrating increases in boundary separation as a function of value conflict.

### Within-system conflict was significantly correlated with between-system conflict

**Fig 5:**
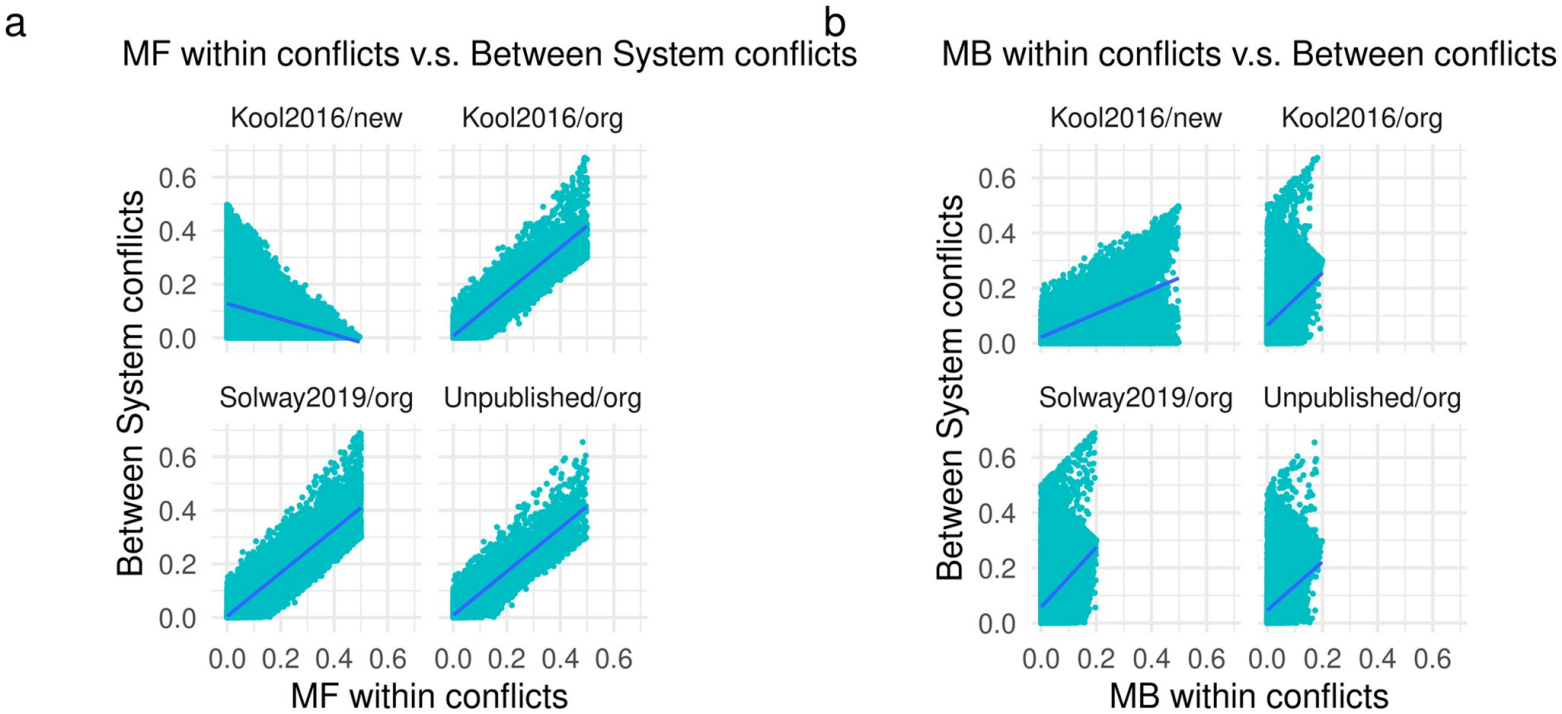
A. Model-free within-system value conflict versus between-system value conflict across all participants and trials for each dataset. B. Model-based within-system value conflict versus between-system value conflict.

Given its definition (Eq 10), value conflict between systems is likely correlated with value conflict within systems (i.e. |(*Q*_*mb*_(*s*, *choice*_1_) − *Q*_*mb*_(*s*, *choice*_2_))| and |(*Q*_*mf*_(*s*, *choice*_1_) − *Q*_*mf*_(*s*, *choice*_2_))|). Fig 5 plots the correlation between value conflict (Eq 10) and each of these terms across all participants and trials for each dataset. The correlation with within-system model-free conflict was substantial in the original version of the task (*ρ* > 0.9 in all three datasets). In contrast, the correlation with within-system model-based conflict was high in the newer task version, although the effect was smaller than the model-free effect in the original task (*ρ* = 0.53).

An alternative explanation for the above results could be that the strength of each system is modulated based on the degree of conflict within the other system alone. A priori it seems less obvious why the preferences of one system would be related to the strength of the other without taking both systems’ values estimates into account. It would be valuable to test this explicitly, although the strength of the correlation between the different forms of conflict makes it difficult to do so with the current task.

**Fig 6:**
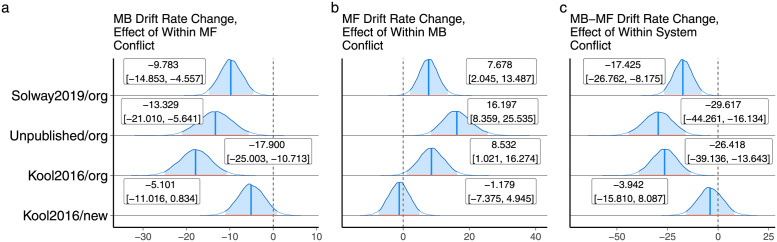
A. The effect of within-system model-free conflict (Q-value differences) on the strength of model-based control. B. The effect of within-system model-based conflict (Q-value differences) on the strength of model-free control. C. Differences between the effects on model-based and model-free control.

**Fig 7:**
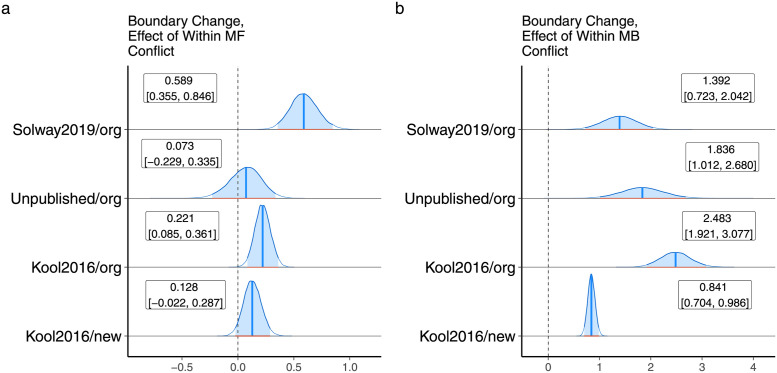
A. The effect of within-system model-free conflict (Q-value differences) on decision boundary separation. B. The effect of within-system model-based conflict on decision boundary separation.

For completeness, we ran separate analyses with within-system conflict effects only. More concretely, this version of the model defined *v*_*mb*_ and *a*_1_ as:

$$\begin{array}{c} v_{mb}=v_{mb,base}+v_{mb,within-conflict}\cdot \left|\left(Q_{mf}\left(s,choice_{1}\right)-Q_{mf}\left(s,choice_{2}\right)\right)\right|, \end{array}$$ (15)

$$\begin{array}{cc} a_{1}=a_{1,base}+ & a_{mb,within-conflict,value}\cdot \left|\left(Q_{mb}\left(s,choice_{1}\right)-Q_{mb}\left(s,choice_{2}\right)\right)\right|+\\ & a_{mf,within-conflict,value}\cdot \left|\left(Q_{mf}\left(s,choice_{1}\right)-Q_{mf}\left(s,choice_{2}\right)\right)\right|, \end{array}$$ (16)

and similarly for *v*_*mf*_. We did not differentiate the presence and absence of action conflict because although between and within-system conflict are related, separating action conflict in this version of the model would have introduced parameters that were perfectly correlated and not identifiable. Figs G-J in S1 Text display parameter recovery results for this model and Figs 6 and 7 display the empirical results, which were similar to the results for between-system conflict. Thus, while our analysis speaks to the importance of non-linear conflict effects, the structure of the task does not allow us to empirically disambiguate the exact form of conflict at play. To complete the parallel analysis, we also ran a version of the model with squared value differences that could be fit in a single step of full Bayesian inference, as we did for the between-system conflict model above. Figs K-L in S1 Text display these results, which were similar to those using the absolute value of the difference.

## Discussion

Across four datasets which encompassed two versions of the well-studied two-step task, we tested whether the strength of model-based and model-free control was modulated by conflict between the two systems. In addition, we tested whether the distance between decision boundaries, representing the relative amount of evidence needed to make a decision (and thus the amount of caution employed), was similarly related to inter-system conflict. We defined *value conflict* to be the absolute difference in predicted action value preference between the two systems, and looked separately at value conflict effects on trials where the two systems disagreed versus agreed on the identity of the best action (with and without value conflict). We found a negative relationship between the strength of model-based control and value conflict in all four datasets on trials without action conflict, as well as in three out of four datasets on trials with action conflict. Moreover, this reduction was generally larger than the corresponding change in model-free control.

Interestingly, the data with the non-significant relationship from the latter group were from the more recent task version where increased model-based control is more rewarding compared to the original version [7]. One interpretation could be that conflict-related reductions in model-based control are attenuated when model-based control is worth it. However, enthusiasm for this interpretation should be tempered for two reasons. First, much of the posterior distribution for the effect is still below zero (Fig 2D). Second, our analysis revealed that there are in fact far fewer trials on average with action conflict in this version of the task (Fig 3C). Thus, a simpler explanation is that questions about action conflict trials in the newer version may be underpowered. We also found in three of the four datasets that value conflict was correlated with larger decision boundaries both in the presence and absence of action conflict. The sole dataset where value conflict related boundary effects did not replicate had the smallest sample size (Table 1), suggesting an issue of power here also as one possible explanation for this failure.

The fact that there were fewer action conflict trials in the newer task version and that value conflict was relatively similar between tasks (Fig 3) may seem counterintuitive on first blush. If the newer version was specifically designed and shown to result in increased payoffs from employing more model-based control [7], it seems that the two systems should differ more in their predictions about which action is more valuable. However, it is important note that model-derived predictions about values translate rather imperfectly to actual outcomes. Kool et al. [7] analyzed this question in detail while designing the new version, looking at correlations between predicted values and outcomes across a range of parameter values in the reinforcement learning model. Correlations were especially poor when outcomes were probabilistic, and were better but still imperfect with deterministic outcomes. Given that questions about conflict rest on differences in the predictions made by the two systems rather than differences in the outcomes received by relying on each system, future simulation work in the spirit of Kool et al. [7] but aimed at understanding task features which maximize differences in predicted values would be fruitful.

Although the findings were consistent across multiple datasets collected in different locations and by different labs, the data do not explain *why* the propensity of model-based control is negatively correlated with conflict, or why decision boundary separation is positively correlated with conflict, especially on trials without action conflict. It is tempting to attempt to interpret these results from a normative perspective. While normative thinking has provided explanatory power in other related contexts [41, 42], and is the basis for a more recent extension of the prominent conflict model we used as a starting point [21], its success in explaining the two-step data in particular is wanting. Even prior to the current work, a salient mystery has been why individuals have engaged in any amount of model-based control in the original version of the task. Doing so does not yield a significantly higher payoff, on average, while increasing the computational cost of the decision [7]. Nevertheless, virtually every study using the task has reported significant levels of model-based control, including all three datasets of the original version reported here [7, 14].

As discussed further below, we advocate more algorithmic thinking and building more detailed decision models constrained by available data. However, we attempt to walk through the logic of a normative interpretation of the conflict results to see how far it can take us. During trials without action conflict, which are the majority of trials (Fig 3C), model-based control can increase the speed and accuracy of decisions (accuracy with respect to both systems) by increasing drift rate. This can be seen in Eq 13: when both systems prefer the same action (have the same sign), they sum together and the result is a larger absolute value of drift rate. This in itself could explain why model-based control is employed in the original version of the task: such an increase in drift rate can potentially reduce opportunity costs by reducing reaction time, even if it does not deliver significant additional reward. However, we found that value conflict was associated with a decreased contribution of model-based control to drift rate, acting in opposition to this beneficial boost. During trials with action conflict, such a reduction may be useful in the original task. With action conflict, the two systems drive drift rate in opposite directions (the signs of the systems’ contributions in Eq 13 are opposite), leading to a decrease in overall drift rate and a potential increase in opportunity costs. Reducing the contribution of model-based control would lessen the degree to which reaction time is unnecessarily prolonged, as it does not yield significantly more reward. However, this logic does not apply to the newer task, where model-based control does result in a higher payoff.

Additional challenges come from the relationship between value conflict and decision boundary separation. While a positive correlation is consistent with previous work on one-step decisions [28, 29, 35], it mostly does not align with a normative interpretation in the present context. In the one-step decision tasks previously studied, increased conflict resulted in reduced overall drift rate (and thus accuracy), which could be compensated for with an increased decision boundary. Such logic might also apply here to trials with action conflict in the newer task version where model-based control is worthwhile and this system is dominant (Table D in S1 Text). An increased boundary would allow the preferences of the model-based system to be further integrated, and compensate for the reduced overall drift rate that results from the disagreement between the systems about which action is better. However, giving additional time to the model-based system is not necessary in the original task, where it would not be worthwhile. Moreover, on trials without action conflict in both tasks, baseline drift rate is actually increased in the direction agreed upon by the two systems because their contributions sum together, as noted above. There is thus nothing for the increased boundary to compensate for in this case.

Rather than attempting to understand these results from a normative perspective, more detailed algorithmic-level modeling work is required. Our approach of using the drift-diffusion model as a link function to translate values computed by the standard hybrid reinforcement learning model commonly applied to this task [4, 7, 13, 14] follows previous work [33]. However, use of the drift-diffusion model in this way is meant to be a statistical approximation; the combined model is not meant to describe the mechanistic cognitive process by which decisions in these tasks evolve. Particularly unrealistic, the combined model assumes that model-based value computations occur in constant time, independent of both the computed values and the one-step rewards on which they are based. Moreover, the model is silent about the specific process by which this computation is performed. In previous work, we explored how more ‘pure’ model-based decisions may be realized by a competitive evidence integration process [34]. In this work, the task was simplified in two ways relative to the current context: all transitions were deterministic (although this was also the case in the newer task version presented here), and more importantly, there was no learning across trials. Stimuli were instead everyday products that participants had to choose between, reducing reliance on model-free contributions. Additional research is necessary in order to develop a more complete process model of model-based control and combined model-free/model-based control. Using reaction time data from the current tasks can help constrain and adjudicate between candidate process models. In addition, the effects of conflict between decision systems presented here place additional constraints on such model development.

Modified forms of the drift-diffusion model have been developed for conflict tasks such as the Stroop and Flanker [43]. One variation of these models assumes that decision making is driven by two processes: one fast and the other slower and more controlled, with the more controlled process beginning later in the decision. Dual process models with potentially differential starting times have also been developed in the context of risky decision making [44]. Such models pose technical challenges with model fitting, although recent progress has been made in this area [43, 45], and it would be of interest to allow for different starting times for the two systems in models of the two-step task. In this context, eye-tracking has suggested that model-based rollouts may actually be what start first, even prior to trial onset [46]. Here we made the more common assumption that the model-based and model-free systems begin working in tandem at the start of the decision and continue in parallel. The standard drift-diffusion model in particular has also likewise been used in prior work with the two-step task [33].

We also found evidence, in three of four datasets, that model-free control was negatively related to action conflict irrespective of the degree of disagreement (i.e. as a function of the binary state). However, this reduction was not significantly different from the change in model-based control in any dataset. In addition, we found that boundary separation was negatively related to action conflict, but while the direction was consistent in all four datasets, the effect was significant in only two of them. These effects were less prominent than those of value conflict. As noted above, however, action conflict trials made up less than 40% of each dataset. In addition, power may be even more of an issue for this binary variable relative to the continuous measure of value conflict. The replicability of these effects thus needs to be further tested, and they likely should not be ignored in the development of mechanistic process models just yet.

Given its definition (Eq 10), between-system conflict was related to within-system conflict. We found this correlation to be substantial, especially in the original version of the task (Fig 5), preventing us from fitting and interpreting both effects within a single model. A priori it is less clear why within-system conflict should modulate the strength of either system (above and beyond its standard linear contribution) without taking into consideration the state of the other system. For example, if the model-based controller had a significant preference for one action over another, it is unclear why the strength of either system should change if the model-free controller also had the same preference. It would of course be valuable to explicitly empirically test this, especially given the fact that the between-system effects, although replicable across distinct datasets, did not align with our predictions. At best, here we could fit non-linear within-system conflict effects separately from between-system conflict, and unsurprisingly given their high correlation, results were similar. Future work is required to develop new tasks that can better differentiate these different forms of conflict.

## Conclusion

In summary, while many previous studies have assumed that model-based and model-free decisions evolve independently and that their predictions sum linearly, our work suggests that the two systems interact in a non-linear fashion, with the propensity of model-based control being influenced by the value conflict between systems. In addition, we found that value conflict was related to the level of caution during decision making. These results were largely consistent across datasets from different labs, which included different populations and data collection methods (online and in-lab). Further work is necessary to construct more realistic process models of decision making that incorporate both systems, and the current results provide additional new constraints that successful process models must capture. In addition, as previous work has shown that OCD and other disorders of compulsivity are associated with a reduction in model-based control [15–17], it would be of interest to explore how conflict effects relate to these individual differences.

## Supporting information

S1 Text Contains tables with detailed parameter estimates and figures for auxiliary analyses described in the main text. (PDF)
