# Supplementary material for: Conflict and competition between model-based and model-free control
Source: PLoS Comput Biol. 2022 May 5;18(5):e1010047. doi: 10.1371/journal.pcbi.1010047 (PMC9070915; doi:10.1371/journal.pcbi.1010047)
Supplement: S1 Text — (PDF) [file pcbi.1010047.s001.pdf]

Supplementary Information

Table A: Solway et al., 2019/orig task Group Parameters

Parameter	Median and CI
First-stage drift rate, model-based, mean	4.41 [2.44, 6.35]
First-stage drift rate, model-based, sd	7.25 [5.82, 8.96]
First-stage drift rate, model-free, mean	3.81 [2.82, 4.81]
First-stage drift rate, model-free, sd	3.76 [3.00, 4.67]
Second stage drift rate, mean	8.48 [7.63, 9.33]
Second stage drift rate, sd	4.38 [3.77, 5.13]
First-stage boundary separation, mean	1.53 [1.48, 1.58]
First-stage boundary separation, sd	0.26 [0.23, 0.31]
Second-stage boundary separation, mean	1.61 [1.57, 1.66]
Second-stage boundary separation, sd	0.24 [0.21, 0.28]
Drift rate perseverance, mean	1.05 [0.89, 1.20]
Drift rate perseverance, sd	0.84 [0.73, 0.97]
Starting point perseverance, mean	-0.31 [-0.37, -0.26]
Starting point perseverance, sd	0.27 [0.23, 0.32]
Non-decision time, mean	0.34 [0.32, 0.35]
Non-decision time, sd	0.09 [0.08, 0.10]
Effect of value conflict without action conflict on model-based drift rate, mean	-16.76 [-24.60, -9.11]
Effect of value conflict without action conflict on model-based drift rate, sd	3.23 [0.16, 10.78]
Main effect of action conflict on model-based drift rate, mean	0.19 [-2.74, 3.06]
Main effect of action conflict on model-based drift rate, sd	4.26 [0.75, 7.14]
Effect of value conflict with action conflict compared to without action conflict on model-based drift rate, mean	4.90 [-8.10, 18.19]
Effect of value conflict with action conflict compared to without action conflict on model-based drift rate, sd	6.98 [0.35, 20.09]
Effect of value conflict without action conflict on model-free drift rate, mean	0.50 [-2.10, 3.09]
Effect of value conflict without action conflict on model-free drift rate, sd	1.16 [0.05, 3.70]
Main effect of action conflict on model-free drift rate, mean	-1.36 [-2.52, -0.19]
Main effect of action conflict on model-free drift rate, sd	0.83 [0.06, 1.74]

cont'd

Effect of value conflict with action conflict compared to without action conflict on model-free drift rate, mean	0.52 [-3.20, 4.35]
Effect of value conflict with action conflict compared to without action conflict on model-free drift rate, sd	1.43 [0.07, 4.06]
Effect of value conflict without action conflict on boundary separation, mean	0.69 [0.46, 0.94]
Effect of value conflict without action conflict on boundary separation, sd	0.71 [0.42, 1.05]
Main effect of action conflict on boundary separation, mean	-0.03 [-0.06, 0.00]
Main effect of action conflict on boundary separation, sd	0.05 [0.01, 0.09]
Effect of value conflict with action conflict compared to without action conflict on boundary separation, mean	-0.27 [-0.49, -0.05]
Effect of value conflict with action conflict compared to without action conflict on boundary separation, sd	0.18 [0.01, 0.42]

Table B: Unpublished/orig task Group Parameters

Parameter	Median and CI
First-stage drift rate, model-based, mean	6.16 [4.21, 8.08]
First-stage drift rate, model-based, sd	5.39 [4.12, 6.99]
First-stage drift rate, model-free, mean	2.65 [1.81, 3.49]
First-stage drift rate, model-free, sd	0.79 [0.18, 1.35]
Second stage drift rate, mean	8.67 [7.31, 10.09]
Second stage drift rate, sd	6.22 [5.26, 7.43]
First-stage boundary separation, mean	1.42 [1.38, 1.46]
First-stage boundary separation, sd	0.17 [0.14, 0.20]
Second-stage boundary separation, mean	1.63 [1.58, 1.67]
Second-stage boundary separation, sd	0.21 [0.18, 0.25]
Drift rate perseverance, mean	0.69 [0.57, 0.80]
Drift rate perseverance, sd	0.52 [0.44, 0.62]
Starting point perseverance, mean	-0.20 [-0.24, -0.15]
Starting point perseverance, sd	0.18 [0.15, 0.23]
Non-decision time, mean	0.22 [0.21, 0.24]
Non-decision time, sd	0.06 [0.05, 0.07]
Effect of value conflict without action conflict on model-based drift rate, mean	-17.67 [-29.59, -5.51]
Effect of value conflict without action conflict on model-based drift rate, sd	3.70 [0.17, 13.05]
Main effect of action conflict on model-based drift rate, mean	0.82 [-2.06, 3.76]
Main effect of action conflict on model-based drift rate, sd	1.31 [0.07, 4.04]
Effect of value conflict with action conflict compared to without action conflict on model-based drift rate, mean	-9.40 [-28.66, 8.77]
Effect of value conflict with action conflict compared to without action conflict on model-based drift rate, sd	7.80 [0.36, 22.73]
Effect of value conflict without action conflict on model-free drift rate, mean	0.29 [-3.06, 3.84]
Effect of value conflict without action conflict on model-free drift rate, sd	1.02 [0.04, 3.62]
Main effect of action conflict on model-free drift rate, mean	-1.53 [-2.91, -0.15]
Main effect of action conflict on model-free drift rate, sd	0.43 [0.02, 1.38]
Effect of value conflict with action conflict compared to without action conflict on model-free drift rate, mean	0.10 [-4.78, 5.13]

cont'd

Effect of value conflict with action conflict compared to without action conflict on model-free drift rate, sd	1.19 [0.06, 3.98]
Effect of value conflict without action conflict on boundary separation, mean	0.17 [-0.19, 0.51]
Effect of value conflict without action conflict on boundary separation, sd	1.02 [0.63, 1.47]
Main effect of action conflict on boundary separation, mean	-0.02 [-0.05, 0.01]
Main effect of action conflict on boundary separation, sd	0.04 [0.00, 0.07]
Effect of value conflict with action conflict compared to without action conflict on boundary separation, mean	-0.22 [-0.54, 0.07]
Effect of value conflict with action conflict compared to without action conflict on boundary separation, sd	0.24 [0.01, 0.66]

Table C: Kool et al., 2016/orig task Group Parameters

Parameter	Median and CI
First-stage drift rate, model-based, mean	7.18 [5.11, 9.24]
First-stage drift rate, model-based, sd	7.07 [5.70, 8.58]
First-stage drift rate, model-free, mean	2.35 [1.55, 3.14]
First-stage drift rate, model-free, sd	1.26 [0.81, 1.69]
Second stage drift rate, mean	7.54 [6.70, 8.39]
Second stage drift rate, sd	5.37 [4.74, 6.11]
First-stage boundary separation, mean	1.27 [1.23, 1.30]
First-stage boundary separation, sd	0.23 [0.20, 0.26]
Second-stage boundary separation, mean	1.60 [1.56, 1.64]
Second-stage boundary separation, sd	0.27 [0.25, 0.31]
Drift rate perseverance, mean	0.98 [0.85, 1.11]
Drift rate perseverance, sd	0.82 [0.73, 0.93]
Starting point perseverance, mean	-0.22 [-0.26, -0.18]
Starting point perseverance, sd	0.23 [0.20, 0.27]
Non-decision time, mean	0.30 [0.29, 0.31]
Non-decision time, sd	0.09 [0.08, 0.10]
Effect of value conflict without action conflict on model-based drift rate, mean	-32.10 [-43.61, -20.85]
Effect of value conflict without action conflict on model-based drift rate, sd	3.68 [0.16, 12.17]
Main effect of action conflict on model-based drift rate, mean	-0.41 [-3.95, 3.18]
Main effect of action conflict on model-based drift rate, sd	5.42 [1.19, 8.38]
Effect of value conflict with action conflict compared to without action conflict on model-based drift rate, mean	12.58 [-6.49, 31.82]
Effect of value conflict with action conflict compared to without action conflict on model-based drift rate, sd	7.86 [0.40, 25.71]
Effect of value conflict without action conflict on model-free drift rate, mean	2.47 [-0.82, 5.73]
Effect of value conflict without action conflict on model-free drift rate, sd	1.01 [0.04, 3.21]
Main effect of action conflict on model-free drift rate, mean	-1.74 [-3.06, -0.43]
Main effect of action conflict on model-free drift rate, sd	1.05 [0.09, 2.01]
Effect of value conflict with action conflict compared to without action conflict on model-free drift rate, mean	0.48 [-4.51, 5.55]

cont'd

Effect of value conflict with action conflict compared to without action conflict on model-free drift rate, sd	1.33 [0.07, 4.37]
Effect of value conflict without action conflict on boundary separation, mean	0.32 [0.17, 0.49]
Effect of value conflict without action conflict on boundary separation, sd	0.46 [0.28, 0.64]
Main effect of action conflict on boundary separation, mean	-0.04 [-0.07, -0.02]
Main effect of action conflict on boundary separation, sd	0.06 [0.02, 0.09]
Effect of value conflict with action conflict compared to without action conflict on boundary separation, mean	-0.10 [-0.29, 0.09]
Effect of value conflict with action conflict compared to without action conflict on boundary separation, sd	0.17 [0.01, 0.42]

Table D: Kool et al., 2016/new task Group Parameters

Parameter	Median and CI
Drift rate, model-based, mean	2.90 [2.34, 3.47]
Drift rate, model-based, sd	1.87 [1.60, 2.16]
Drift rate, model-free, mean	1.47 [0.85, 2.09]
Drift rate, model-free, sd	1.93 [1.55, 2.33]
Boundary separation, mean	1.56 [1.52, 1.60]
Boundary separation, sd	0.25 [0.22, 0.28]
Drift rate perseverance, mean	0.39 [0.34, 0.43]
Drift rate perseverance, sd	0.23 [0.20, 0.27]
Starting point perseverance, mean	-0.11 [-0.14, -0.08]
Starting point perseverance, sd	0.09 [0.06, 0.12]
Non-decision time, mean	0.38 [0.36, 0.39]
Non-decision time, sd	0.11 [0.10, 0.13]
Effect of value conflict without action conflict on model-based drift rate, mean	-4.83 [-6.56, -3.12]
Effect of value conflict without action conflict on model-based drift rate, sd	1.45 [0.08, 3.25]
Main effect of action conflict on model-based drift rate, mean	-1.03 [-2.28, 0.22]
Main effect of action conflict on model-based drift rate, sd	1.76 [0.54, 2.48]
Effect of value conflict with action conflict compared to without action conflict on model-based drift rate, mean	2.24 [-2.14, 6.63]
Effect of value conflict with action conflict compared to without action conflict on model-based drift rate, sd	2.65 [0.14, 6.68]
Effect of value conflict without action conflict on model-free drift rate, mean	-0.74 [-4.23, 2.77]
Effect of value conflict without action conflict on model-free drift rate, sd	2.37 [0.09, 7.34]
Main effect of action conflict on model-free drift rate, mean	1.13 [-2.05, 4.33]
Main effect of action conflict on model-free drift rate, sd	5.69 [3.19, 7.99]
Effect of value conflict with action conflict compared to without action conflict on model-free drift rate, mean	11.66 [-6.47, 29.69]
Effect of value conflict with action conflict compared to without action conflict on model-free drift rate, sd	7.07 [0.34, 23.06]
Effect of value conflict without action conflict on boundary separation, mean	0.69 [0.55, 0.84]

cont'd

Effect of value conflict without action conflict on boundary separation, sd	0.39 [0.15, 0.56]
Main effect of action conflict on boundary separation, mean	-0.06 [-0.10, -0.02]
Main effect of action conflict on boundary separation, sd	0.04 [0.00, 0.09]
Effect of value conflict with action conflict compared to without action conflict on boundary separation, mean	-0.08 [-0.38, 0.23]
Effect of value conflict with action conflict compared to without action conflict on boundary separation, sd	0.27 [0.01, 0.62]

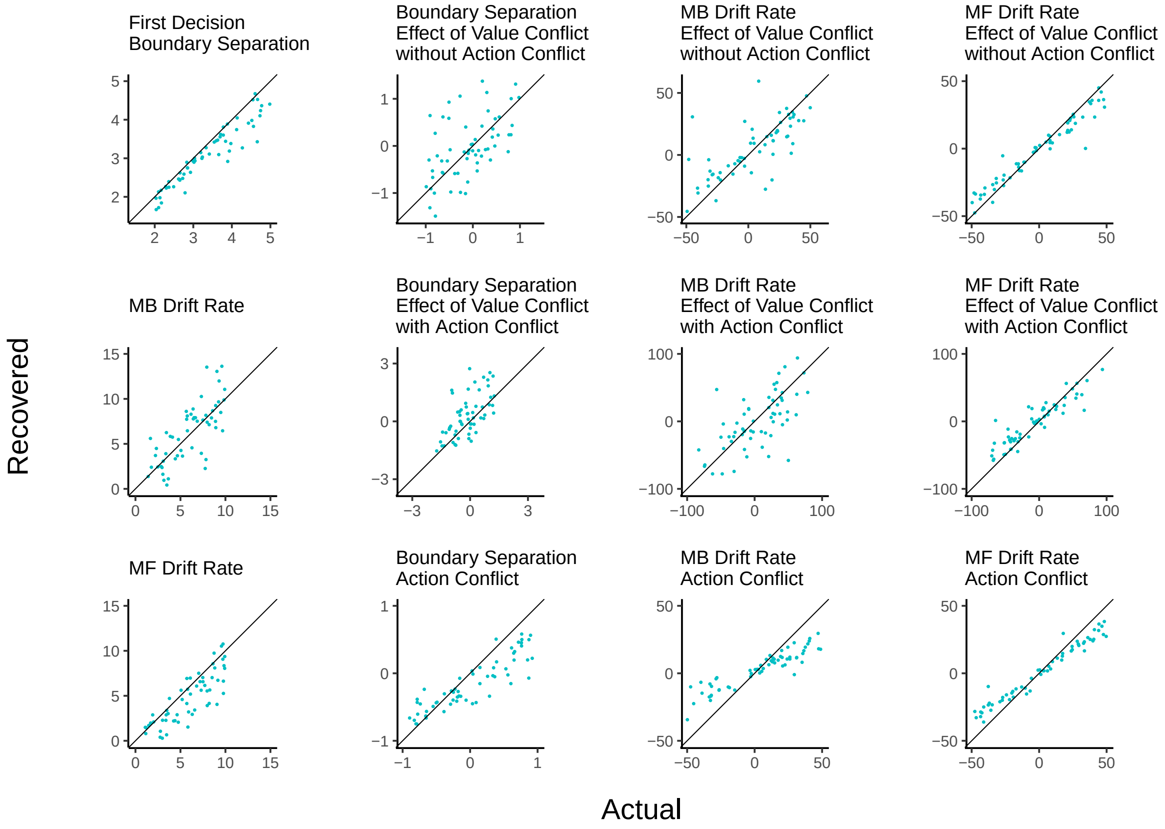

Figure A: Results of a simulation study testing the ability to recover parameters of interest in the between-system conflict model. 60 synthetic datasets were simulated using the participant and trial numbers from Solway et al., 2019. For reference, the diagonal line is the identity line with slope 1, intercept 0.

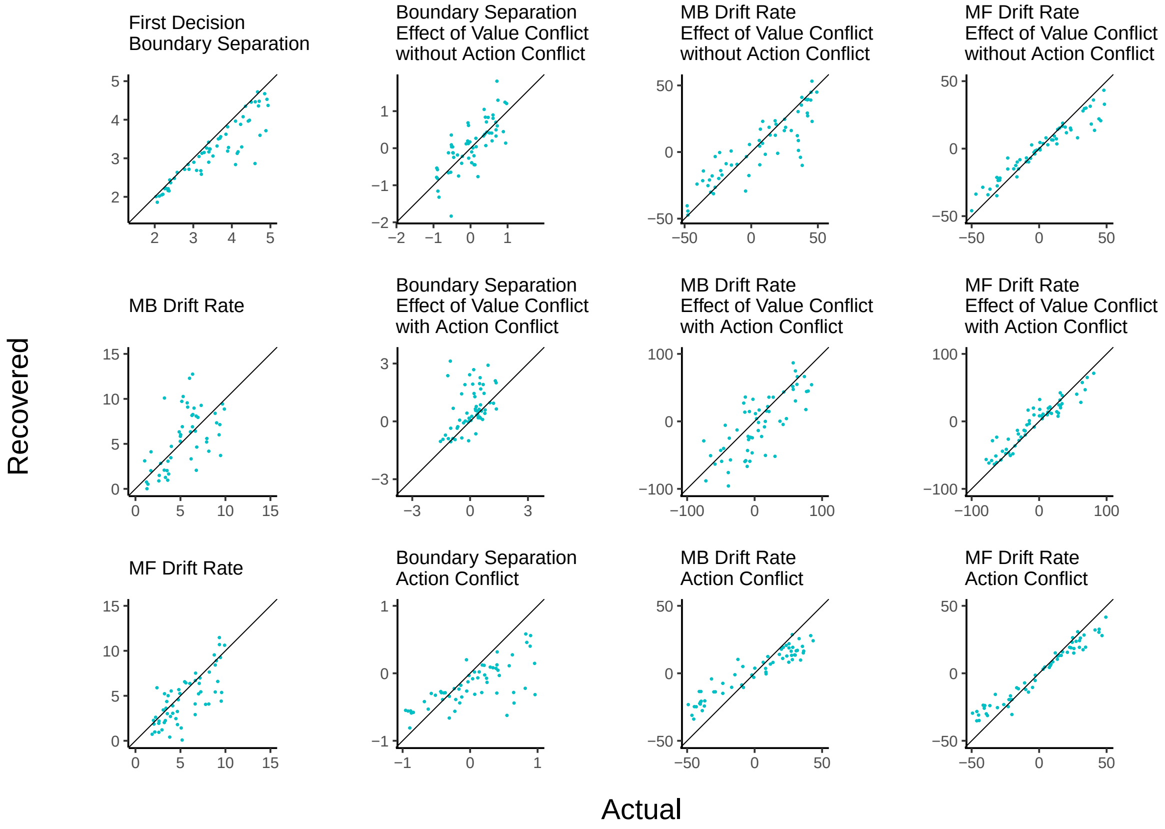

Figure B: Results of a simulation study testing the ability to recover parameters of interest in the between-system conflict model. 60 synthetic datasets were simulated using the participant and trial numbers from the previously unpublished dataset we analyzed. For reference, the diagonal line is the identity line with slope 1, intercept 0.

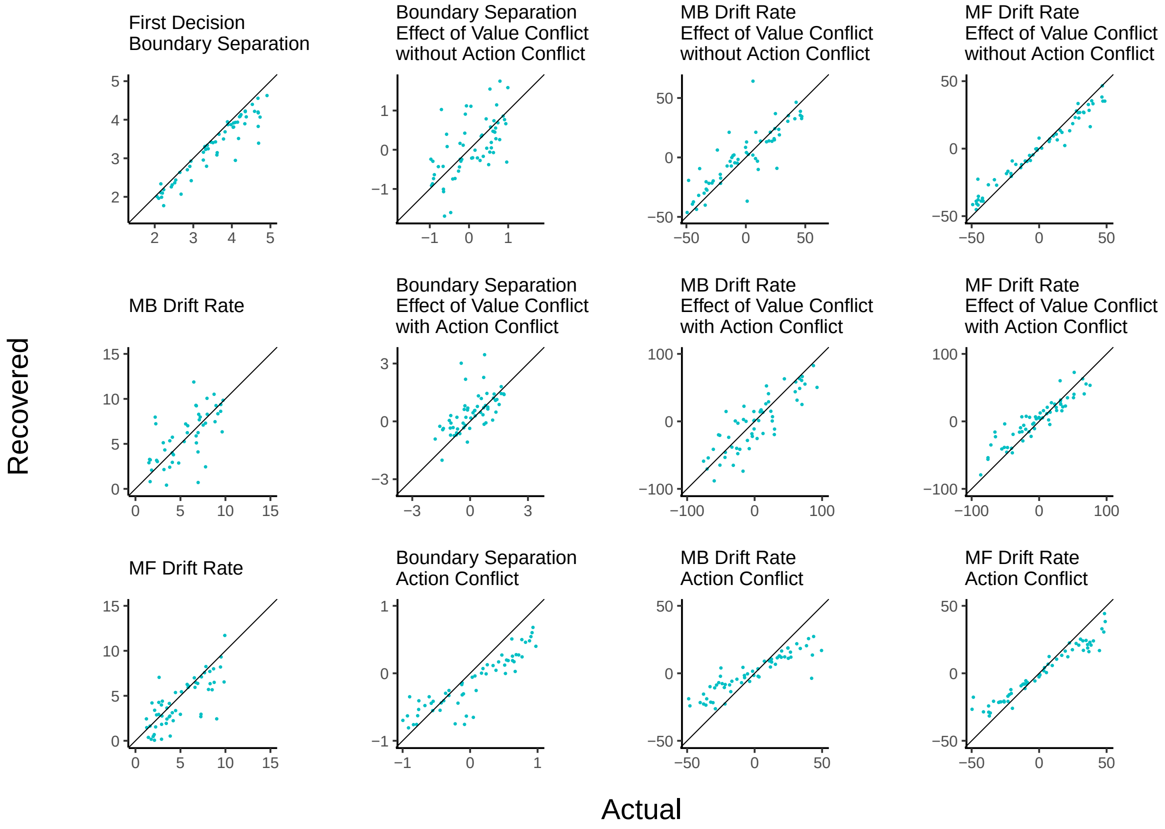

Figure C: Results of a simulation study testing the ability to recover parameters of interest in the between-system conflict model. 60 synthetic datasets were simulated using the participant and trial numbers used in the run of the original task in Kool et al., 2016. For reference, the diagonal line is the identity line with slope 1, intercept 0.

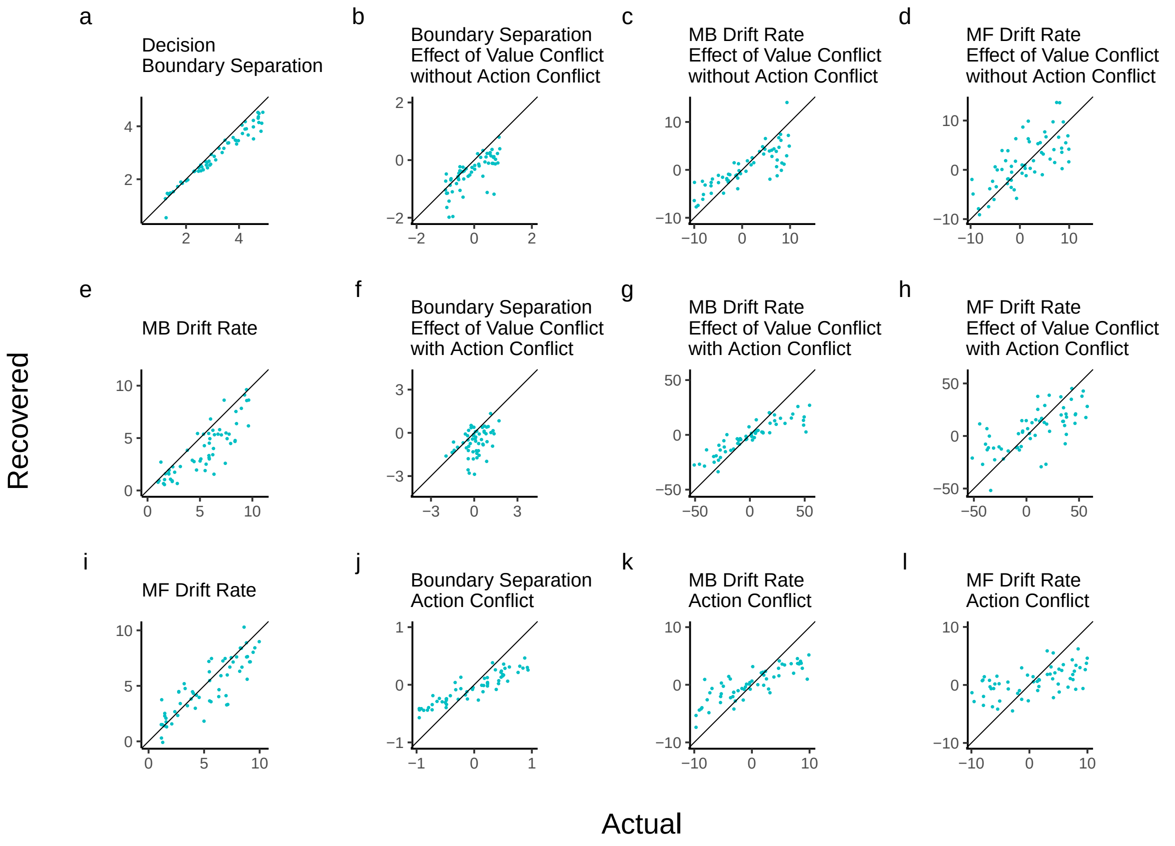

Figure D: Results of a simulation study testing the ability to recover parameters of interest in the between-system conflict model. 60 synthetic datasets of the new task version were simulated using the participant and trial numbers used in the run of the new task in Kool et al., 2016. For reference, the diagonal line is the identity line with slope 1, intercept 0.

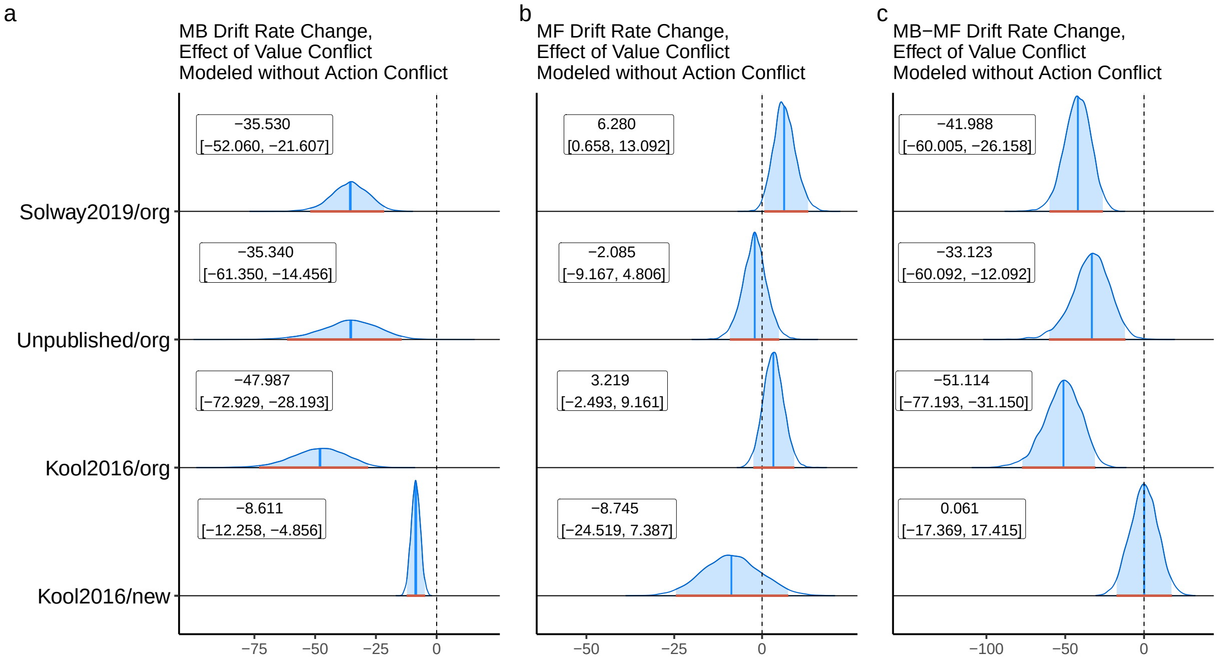

Figure E: The effects of between-system value conflict on each system in a modified model which could be fit in a single model fitting step. Value conflict was calculated as the squared difference between the systems' predictions, and action conflict was excluded, ensuring a differentiable posterior that Stan could efficiently sample without having to fit the learning component and the drift-diffusion components separately.

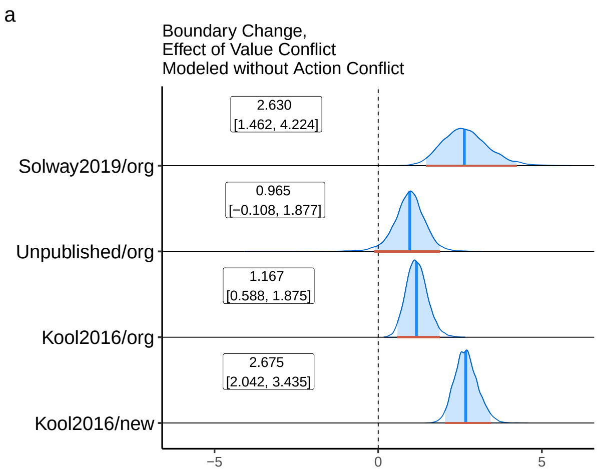

Figure F: The effect of between-system value conflict on boundary separation in a modified model which could be fit in a single model fitting step (see also Fig E).

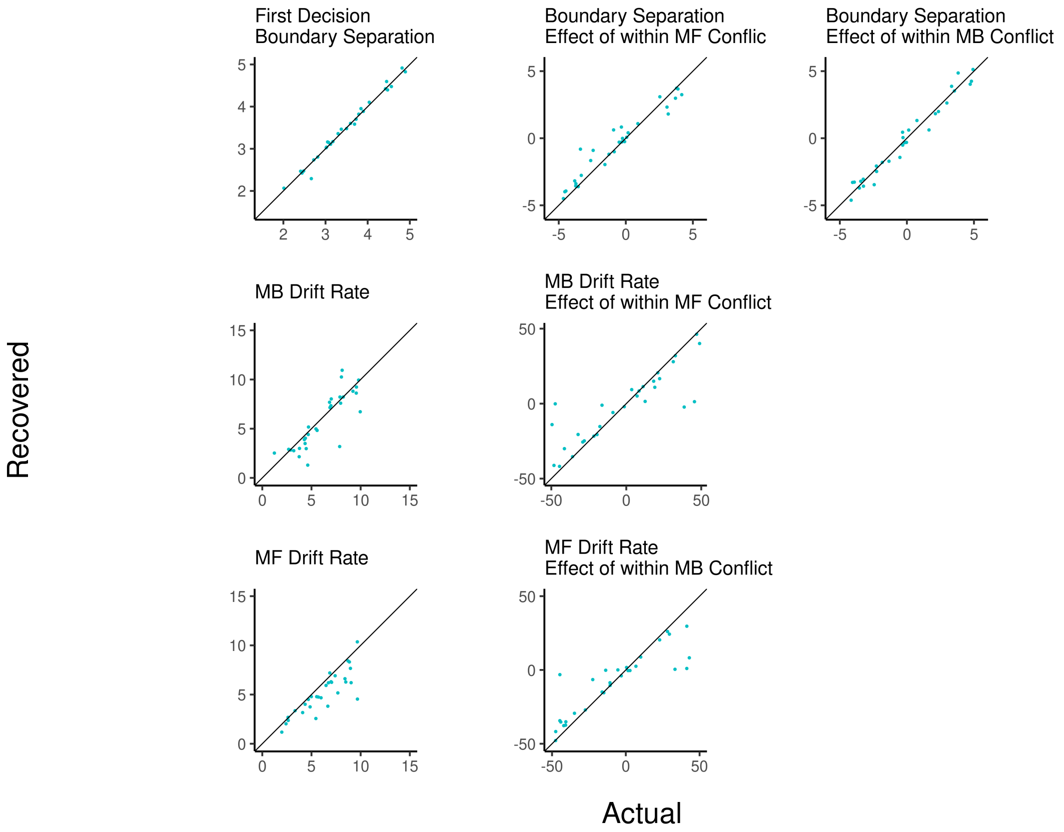

Figure G: Results of a simulation study testing the ability to recover parameters of interest in the within-system conflict model. 30 synthetic datasets were simulated using the participant and trial numbers from Solway et al., 2019. For reference, the diagonal line is the identity line with slope 1, intercept 0.

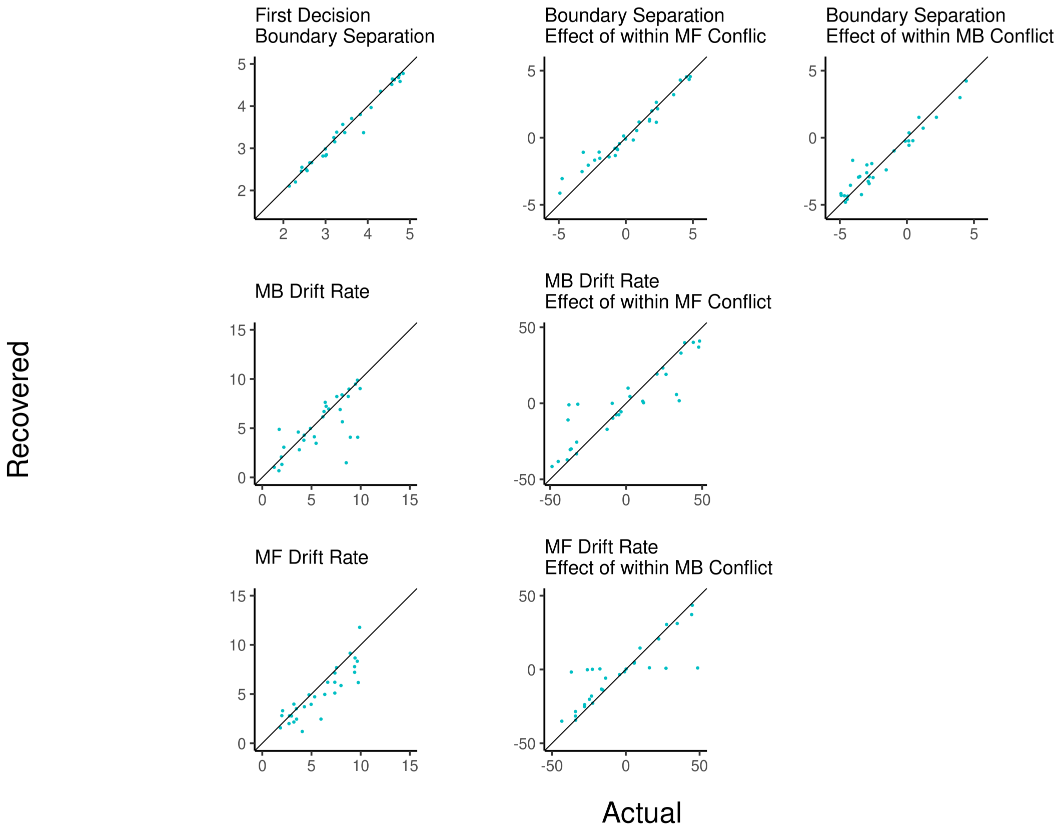

Figure H: Results of a simulation study testing the ability to recover parameters of interest in the within-system conflict model. 30 synthetic datasets were simulated using the participant and trial numbers from the previously unpublished dataset we analyzed. For reference, the diagonal line is the identity line with slope 1, intercept 0.

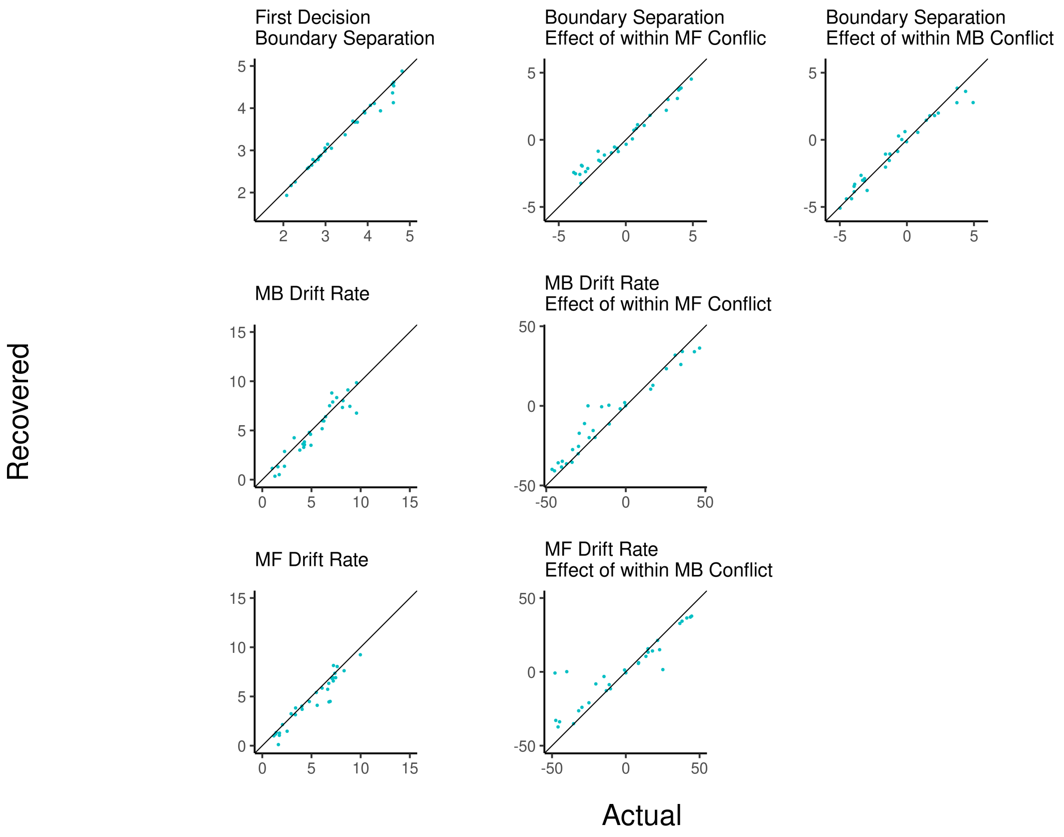

Figure I: Results of a simulation study testing the ability to recover parameters of interest in the within-system conflict model. 30 synthetic datasets were simulated using the participant and trial numbers used in the run of the original task in Kool et al., 2016. For reference, the diagonal line is the identity line with slope 1, intercept 0.

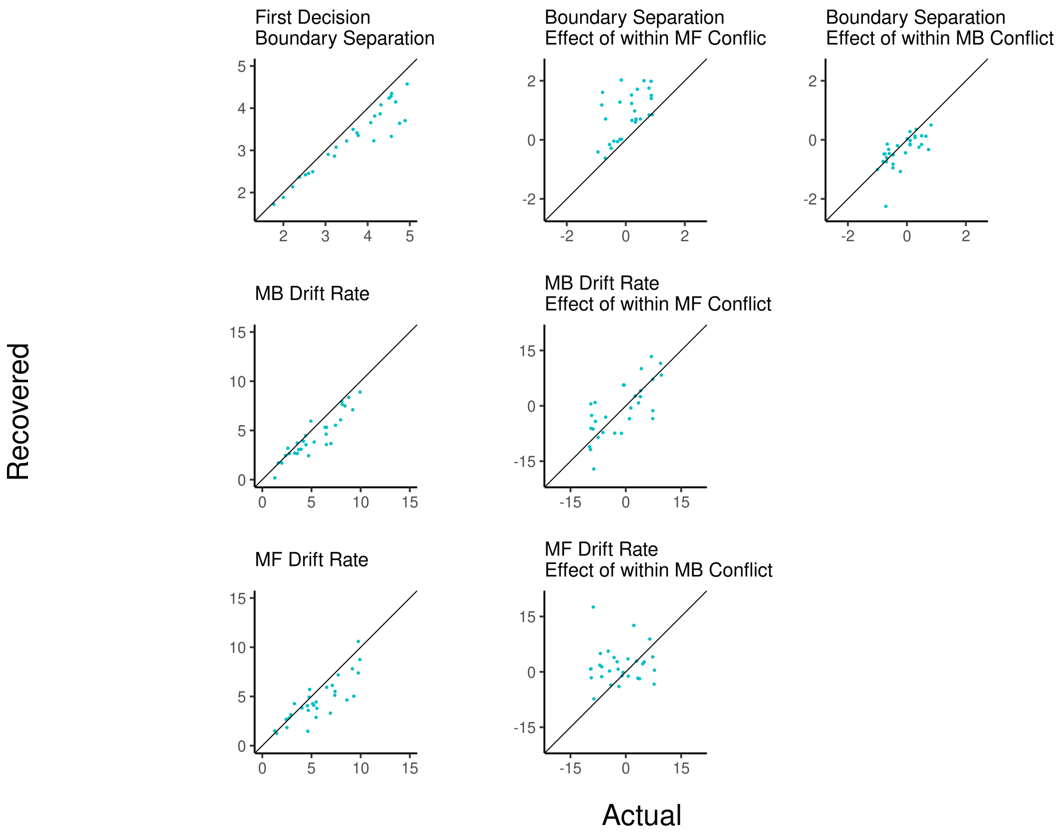

Figure J: Results of a simulation study testing the ability to recover parameters of interest in the within-system conflict model. 30 synthetic datasets of the new task version were simulated using the participant and trial numbers used in the run of the new task in Kool et al., 2016. For reference, the diagonal line is the identity line with slope 1, intercept 0.

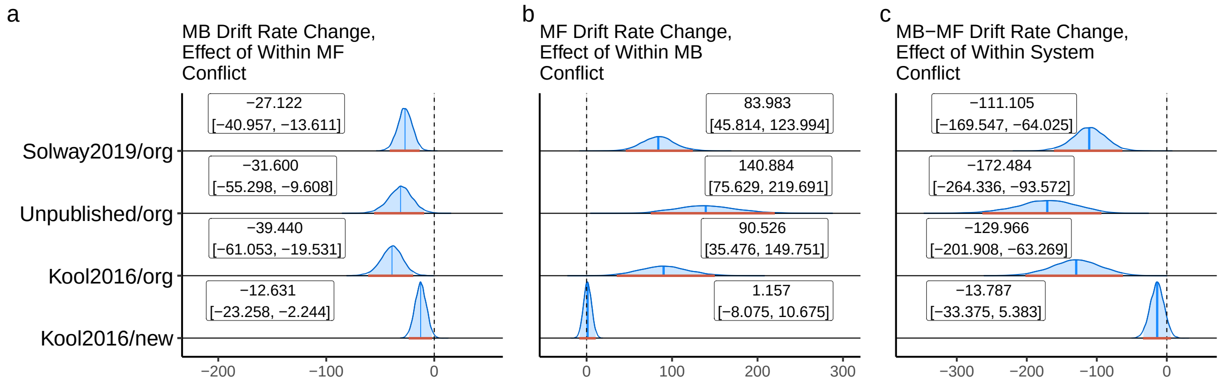

Figure K: The effects of within-system conflict on the strength of the other system in a modified model where conflict was calculated based on the squared difference between action values instead of the absolute difference. These results parallel those in Fig E for between-system conflict.

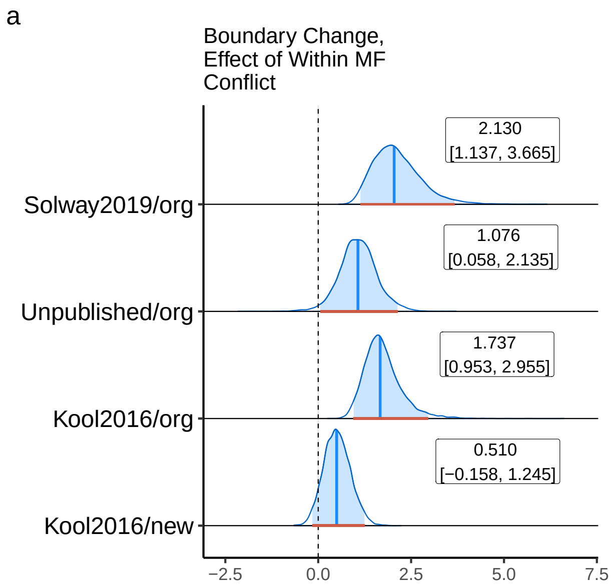

Figure L: The effects of within-system conflict on boundary separation in a modified model where conflict was calculated based on the squared difference between action values instead of the absolute difference. These results parallel those in Fig F for between-system conflict.
